# Sini san regulates intestinal flora and short-chain fatty acids to ameliorate hepatocyte apoptosis and relieve CCl_4_-induced liver fibrosis in mice

**DOI:** 10.3389/fphar.2024.1408459

**Published:** 2024-08-30

**Authors:** Qiong Wu, Fangsi Zhu, Yu Yao, Luyun Chen, Yijie Ding, Yong Su, Chaoliang Ge

**Affiliations:** ^1^ School of Pharmacy, The First Affiliated Hospital of Anhui Medical University, Anhui Medical University, Hefei, China; ^2^ Department of Pharmacy, Anhui No. 2 Provincial People’s Hospital, Hefei, Anhui, China; ^3^ Department of Pharmacy, The First Affiliated Hospital of Anhui Medical University, Hefei, Anhui, China

**Keywords:** Si-Ni-San, intestinal flora, short-chain fatty acids, hepatocyte apoptosis, liver fibrosis, pseudo germ-free mice

## Abstract

**Introduction:**

Si-Ni-San (SNS), a traditional Chinese medicine, is effective in treating liver fibrosis with an unclear mechanism. Although disturbance of intestinal flora and the subsequent secretion of short-chain fatty acids (SCFAs) is suggested to be involved in the progression of liver fibrosis, whether SNS produces the anti-fibrosis effect through the regulation of intestinal flora and SCFAs remains unclear.

**Methods:**

In the current study, carbon tetrachloride (CCl_4_)-treated mice were dosed with SNS to examine the anti-fibrotic effects and the involved mechanism. Biochemical parameters, histological staining, and analyses of fibrotic gene expression were used to evaluate the anti-fibrotic effect of SNS, while intestinal flora and SCFA content were determined by 16S rRNA and LC–MS to evaluate the mechanism.

**Results:**

*In vivo* results showed that SNS improved liver function, reduced hepatocyte apoptosis and FFAR2/3 expression, and restored intestinal dysbiosis and reduced PA, BA, and IsA levels. *In vitro* experiments showed that PA, BA, and IsA exacerbated TNF-α-induced HepG2 apoptosis. Notably, the protective effects of SNS were compromised in pseudo-sterile mice.

**Discussion:**

In conclusion, our experimental results suggest that the disturbance in intestinal flora results in elevated SCFA levels, which further exacerbates hepatocyte apoptosis in liver fibrosis, while SNS suppresses CCl_4_-induced liver fibrosis at least partially by reinstating intestinal flora homeostasis and reducing SCFA levels.

## 1 Introduction

Liver fibrosis is a pathophysiological process caused by chronic liver injuries (Kisseleva and Brenner, 2021). The long-term progression of liver fibrosis can lead to cirrhosis (Caligiuri et al., 2021), liver failure, and portal hypertension (Bataller and Brenner, 2005). Hepatocyte apoptosis plays a key role in the progression of various liver injuries, suggesting the importance of anti-apoptosis for the treatment of liver fibrosis (Filliol et al., 2017; Meng et al., 2018; Wu et al., 2019), while a close connection between hepatocyte apoptosis and intestinal flora has been extensively found (Zhao et al., 2022; Lin et al., 2023b; Song et al., 2023; Yuan et al., 2023). However, the options of pharmacological treatment that targets intestinal flora for ameliorating hepatocyte apoptosis remain to be explored.

The gut–liver axis serves as a critical factor in maintaining liver health. Impaired intestinal barrier function and dysregulated intestinal flora promote the influx of intestinal microorganisms, antigens, or toxic metabolites into the liver, thereby triggering direct liver injury (Li Y-G. et al., 2022). The dysbiosis could also disrupt the liver immune microenvironment, leading to the release of inflammatory factors, which further accelerates the progression of liver fibrosis (Huang et al., 2023), while restoration of intestinal flora balance is believed to effectively mitigate liver fibrosis (Gui et al., 2021), suggesting intestinal flora to be a potential target for treating liver fibrosis.

Short-chain fatty acids (SCFAs), produced by intestinal microbiota metabolism, are saturated fatty acids with chain lengths of 1–6 carbon atoms. They are primarily produced in the colon. Acetic, propionic, and butyric acids are present in 95% of the total SCFAs, with less amounts of valeric, isovaleric, caproic, and isocaproic acids (Morrison and Preston, 2016; Sun et al., 2017; Tan et al., 2023). Studies have shown that disorders of intestinal flora can accelerate liver fibrosis (Le Roy et al., 2013; Hu et al., 2021; Awoniyi et al., 2023), while microbial SCFAs are shown to induce the apoptosis of various cells through G protein-coupled receptors (GRP41 and GRP43, also known as free fatty acid receptors FFAR3 and FFAR2) (Kimura et al., 2001; Shi et al., 2014), suggesting that SCFAs potentially accelerate liver fibrosis by exacerbating hepatocyte apoptosis, which deserves further investigation.

Si-Ni-San (SNS) is a traditional Chinese medicine that is used in the clinical treatment of liver fibrosis, and its main ingredients include *Bupleurum falcatum* L. (Apiaceae), fruit of *Citrus aurantium* L. (Rutaceae), root of *Paeonia lactiflora* Pall. (Paeoniaceae), and rhizome of *Glycyrrhiza uralensis* Fisch. (Leguminosae). Our previous studies demonstrated that SNS improved CCl_4_-induced liver fibrosis and ameliorated hepatocyte apoptosis (Jiang et al., 2023). However, how intestinal flora and its production of SCFAs contributes to the anti-apoptosis effects of SNS during the treatment of CCl_4_-induced liver fibrosis is still not reported.

In this study, we hypothesized that intestinal flora-produced SCFAs accelerated hepatocyte apoptosis, while the effect of SNS in ameliorating hepatocyte apoptosis was related to its modulation on the intestinal flora and its production of SCFAs. We explored the effects of SNS on intestinal flora and SCFAs in CCl_4_-treated mice and examined the contribution of intestinal flora to the anti-fibrosis effect of SNS by using pseudo-sterile mice.

## 2 Materials and methods

### 2.1 Preparation of SNS decoction

The SNS decoction was prepared as described in our previous report (Jiang et al., 2023). SNS consists of *Bupleurum falcatum* L. [Apiaceae], *Citrus × aurantium* f. aurantium [Rutaceae], *Paeonia lactiflora* Pall. [Paeoniaceae], and *Glycyrrhiza uralensis* Fisch. [Leguminosae, Glycyrrhiza uralensis Fisch.ex DC] in a ratio of 1:1:1:1. SNS was decocted with 10 times the volume of distilled water for 1.5 h. The residue was then added to eight times the volume of distilled water for a second decoction. Finally, the residue was added to six times the volume of distilled water and decocted for 0.5 h. The solution of three decoctions were combined and filtered through a sterile gauze, the residue was removed, and the liquid was subsequently concentrated to 0.31 g/mL. SNS decoctions was freshly stored in a −20°C freezer for up to 2 days before use. The content determination of the SNS decoction has been published in our previous study (Jiang et al., 2023).

### 2.2 Reagents and chemicals

Olive oil (Cat. S30503) was purchased from Yuanye Bio-Technology Co., Ltd. (Shanghai, China). Carbon tetrachloride (CCl_4_) (Cat. C805329) was purchased from Macklin (Shanghai China). Vancomycin (Cat. V10549), neomycin sulfate (Cat. N109017), ampicillin (Cat. A105483), and metronidazole (Cat. M109874) were purchased from Shanghai Aladdin Biochemical Technology Co., Ltd. (Shanghai, China). Aspartate aminotransferase (AST, Cat. C010-2-1), alanine aminotransferase (ALT, Cat. C009-2-1), and alkaline phosphatase (ALP, Cat. A059-2-2) kits were purchased from Nanjing Jiancheng Bioengineering Institute (Nanjing China). Recombinant human TNF-α (Cat. 031825) was purchased from PeproTech (Shanghai, China). Actinomycin D (Cat. HY-17559) was purchased from MedChemExpress (Shanghai, China). Cell Counting kit-8 (Cat.RK001099) was purchased from Beijing Medical Technology Co., Ltd. (Anhui, China). Anti-caspase-3 (Cat. 19677-1-AP), anti-FFAR2 (Cat. 19952-1-AP), and anti-FFAR3 (Cat. 66811-1-Ig) antibodies were purchased from Proteintech (Wuhan, China). TRIzol reagent (Cat. 15596026) was obtained from Invitrogen (Carlsbad, CA, United States). SYBR Green Premix (Cat. AG11701) and Evo M-MLV RT Premix (Cat. AG11706) were purchased from Accurate Biology (Hunan, China). The annexin V-FITC/PI double staining apoptosis detection kit (BB-4101) was purchased from Bibo Biotech (Shanghai, China). Sodium butyrate (Cat. IS0190) was purchased from Solarbio (Beijing, China). Sodium propionate (Cat. Abs42086166) and isovaleric acid (Cat. Abs47050746) were purchased from Shanghai Universal Biotech Co., Ltd. (Shanghai, China).

### 2.3 Animal and experimental design

Male C57BL/6J mice, 6–8 weeks old and weighing between 16 and 22 g, were purchased from Hangzhou Zhiyuan Laboratory Animal Technology Co. (SCXK (Zhe) 2019-0004) and maintained in the Anhui Academy of Medical Sciences. The mice were bred in an environmentally controlled room (temperature, 22–24°C; relative humidity, 50%–60%) under a 12-h light/dark cycle with unlimited access to food and water. After adapting for 1 week, the age and body weight-matched mice were divided into three groups (n = 6): the control group (NC group), CCl_4_ group, and CCl_4_ group administered SNS (CCl_4_+SNS group). The mice were injected intraperitoneally with CCl_4_ (0.5 mL/kg, dissolved in olive oil) three times a week for 6 weeks to establish a liver fibrosis model, and NC group mice were injected with equal amounts of lysates. After 2 weeks of CCl_4_ injection, the SNS group was administered with 6.2 g/kg of SNS (20 mL/kg) once daily, based on the optimal dose determined in our previous experiments. After 6 weeks, mice were anesthetized using intraperitoneal injections of tribromoethanol (300 mg/kg) and executed by cervical dislocation after orbital blood extraction (once; 0.2 mL) when they were unresponsive to mild stimuli.

Male C57BL/6J mice, 6–8 weeks old and weighing between 16 and 22 g, were used to construct pseudo-sterile mice and housed under the same conditions as described above. After a 1-week adaptive feeding, the age- and body weight-matched mice were divided into six groups (n = 6): NC group, CCl_4_ group, CCl_4_ + SNS group, pseudo-sterile group (PG), PG + CCl_4_ group, and PG + CCl_4_ + SNS group. Pseudo-sterile mice were prepared using a previously described method: antibiotics (0.5 g/L vancomycin, 1 g/L neomycin sulfate, 1 g/L ampicillin, and 1 g/L metronidazole) were added to the drinking water, which was changed daily for 6 weeks (Chambers et al., 2022). The dosing method for CCl_4_ and SNS was the same as described above. All experiments were conducted under the approval of the Ethics Committee of Anhui Medical University (LLSC20210242).

### 2.4 Biochemical analysis

Mice serum was centrifuged at room temperature at 3,000 rpm for 15 min, and the supernatant was collected and stored at −80°C. Levels of ALT, AST, and ALP were measured using automated chemistry kits.

### 2.5 Histological analysis

Liver tissue was fixed with 10% formalin solution and embedded in paraffin, cut at 5 μm, and triple-stained with hematoxylin–eosin (H&E) and Masson according to standard procedures. Three independent fields of view were randomly selected from the specimen at ×20 magnification. Images were obtained through Pannoramic MIDI (3DHISTECH, Hungary).

### 2.6 Terminal deoxynucleotidyl transferase dUTP nick end labeling (TUNEL) staining

After dewaxing, liver tissue sections were incubated sequentially using 20 μg/mL of DNase-free protease and a TUNEL assay solution. The reaction was subsequently terminated by a labeled reaction stop solution, and streptavidin–HRP working solution was added. After washing, the DAB chromogenic solution was added, and hematoxylin staining was performed. Finally, sections were graded using a 1% ethanol solution of hydrochloric acid and stained blue using a lithium carbonate solution. Images were obtained using Pannoramic MIDI (3DHISTECH, Hungary).

### 2.7 Immunofluorescence detection

Immunofluorescence staining was used to determine the expression of α-smooth muscle actin (α-SMA), FFAR2, and FFAR3. Briefly, after dewaxing and antigen retrieval, tissue sections were incubated with α-SMA, FFAR2, and FFAR3 primary antibodies overnight and then stained with appropriate secondary antibodies for 2 h. Finally, sections were incubated with 4′,6-diamidino-2-phenylindole (DAPI) for 10 min. Images were obtained by Pannoramic MIDI (3DHISTECH, Hungary).

### 2.8 16S rRNA gene sequencing

The mouse feces were collected the day before euthanasia and put into a new sterile tube. The fecal samples were submitted to Oyi Biotech for 16s intestinal flora sequencing, fecal DNA was extracted using the FastDNA SPIN Kit (MP Biomedicals, Santa Ana, CA, United States), and the V16 region of the 4S rRNA gene was amplified using dual index primers. Reads were processed and quality-filtered using Quantitative Insights into Microbial Ecology (QIIME) (v1.9.1) software, and chimera-free sequences were compared using the SILVA database (http://www.arb-silva.de) at 97% identity sex threshold. The amplicons were then sequenced using Illumina MiSeq and MiSeq Reagent Kit V3 (Illumina, San Diego, CA, United States). Paired-end sequencing was performed using the Illumina MiSeq platform. Each deduplicated feature sequence (ASV) was generated by the DADA2 method after denoising, splicing, and other quality control operations; α-diversity is determined using diversity indices such as Shannon and Simpson; PCA, LEfSe, LDA, heatmap, etc. are performed using R software analysis to visualize β-diversity.

### 2.9 SCFA quantification analysis

The serum was submitted to Luming Biotechnology Company, and SCFAs were quantified via liquid chromatography–tandem mass spectrometry (LC–MS/MS). Briefly, 80 μL of the sample was taken, 80 μL of 50% acetonitrile–water solution (v/v) was added; then ultrasonic extraction in an ice–water bath was performed for 10 min, and samples were centrifuged. The clear supernatant liquid is transferred to the injection vial, derivatized, separated by UPLC, and detected via MS/MS.

### 2.10 Western blot analysis

Proteins were extracted from liver tissues using RIPA buffer containing protease inhibitors and phenylmethylsulfonyl fluoride (PMSF). Proteins were separated using sodium dodecyl sulfate polyacrylamide (SDS-PAGE) gels and transferred to polyvinylidene fluoride (PVDF) membranes. Proteins were blocked using milk for 2 h at room temperature and incubated with α-SMA, caspase-3, FFAR2, FFAR3, collagen-I (Col-I), apoptosis regulator Bax (Bax), and apoptosis regulator Bcl-2 (Bcl-2) primary antibodies at a ratio of 1:1000 at 4°C overnight. After incubation with secondary antibodies for 1 h, luminescence imaging was performed.

### 2.11 RT-qPCR

Total RNA was first extracted from liver tissue using TRIzol reagent, and reverse transcription was performed using the PrimeScript™ RT kit. Real-time fluorescence quantitative PCR was performed using the SYBR green real-time fluorescence quantitative PCR kit. β-actin was used as a normalization control. Relative expression levels were assessed using the 2^−ΔΔ^CT method. The primer sequences used in this study are included in Supplementary Table S1.

### 2.12 Cell experiments

The human liver cancer cell HepG2 purchased from Procell Life Sciences (Wuhan, China) was maintained in Dulbecco’s modified Eagle’s medium (DMEM) supplemented with 10% fetal bovine serum and 1% penicillin–streptomycin in an incubator at 37°C and 5% CO_2_. Cells were incubated with actinomycin D (0.2 μM) for 30 min, after which the supernatant was discarded. The fresh medium containing TNF-α (20 ng/mL) was then added to induce apoptosis over a 24-h period. To assess the effects of propionic acid, butyric acid, and isovaleric acid, these compounds were added simultaneously with TNF-α.

### 2.13 Statistical analysis

The data are presented as the mean ± standard error of the mean. Group differences were analyzed using one-way analysis of variance (ANOVA), followed by Tukey’s test for normally distributed data, and performed using GraphPad Prism 8.0 software. For non-normally distributed data, the Kruskal–Wallis test was conducted using SPSS 17. The chi-square test was employed to compare categorical data between groups. Each experiment was repeated three times, and *P* < 0.05 was considered statistically significant. Principal component analysis (PCA) and heatmap analyses were performed using the MetaboAnalyst 5.0 online platform (www.metaboanalyst.ca).

## 3 Results

### 3.1 SNS ameliorated CCl_4_-induced liver fibrosis and hepatocyte apoptosis in mice

In this experiment, we first examined the effect of SNS on the liver function of CCl_4_-treated mice, and the results showed that SNS significantly reduced the CCl_4_-induced elevation of ALT, AST, and ALP (Supplementary Figure S1A). H&E staining demonstrated that SNS ameliorated hepatocyte cell death and the infiltration of inflammatory cells in CCl_4_-treated mice. Masson’s staining showed that SNS reduced pseudo-leaflet and fibrotic septum formation (Supplementary Figure S1B), while this anti-fibrosis effect of SNS was further confirmed by the expression of α-SMA and collagen-1 (Supplementary Figures S2A–C). These results demonstrate that SNS protects against CCl_4_-induced fibrosis.

Apoptosis in the liver tissue was further measured using the TUNEL assay. The results showed that a large amount of positive cells were observed in the CCl_4_-induced model mice, which was significantly reduced by SNS treatment (Supplementary Figure S3A), suggesting that SNS could suppress CCl_4_-induced hepatocyte apoptosis. Western blotting was used to measure the protein expression of the apoptosis-related markers Bcl-2, Bax, and caspase-3. In CCl_4_-treated mice, SNS reduced the expression of caspase-3 and Bax/Bcl-2 (Supplementary Figure S3B), which was consistent with the results of TUNEL assay. These data collectively support the protective effects of SNS against CCl4-induced liver fibrosis and hepatocyte apoptosis.

### 3.2 SNS restored CCl_4_-induced intestinal microbiota disturbance in mice

**FIGURE 1:**
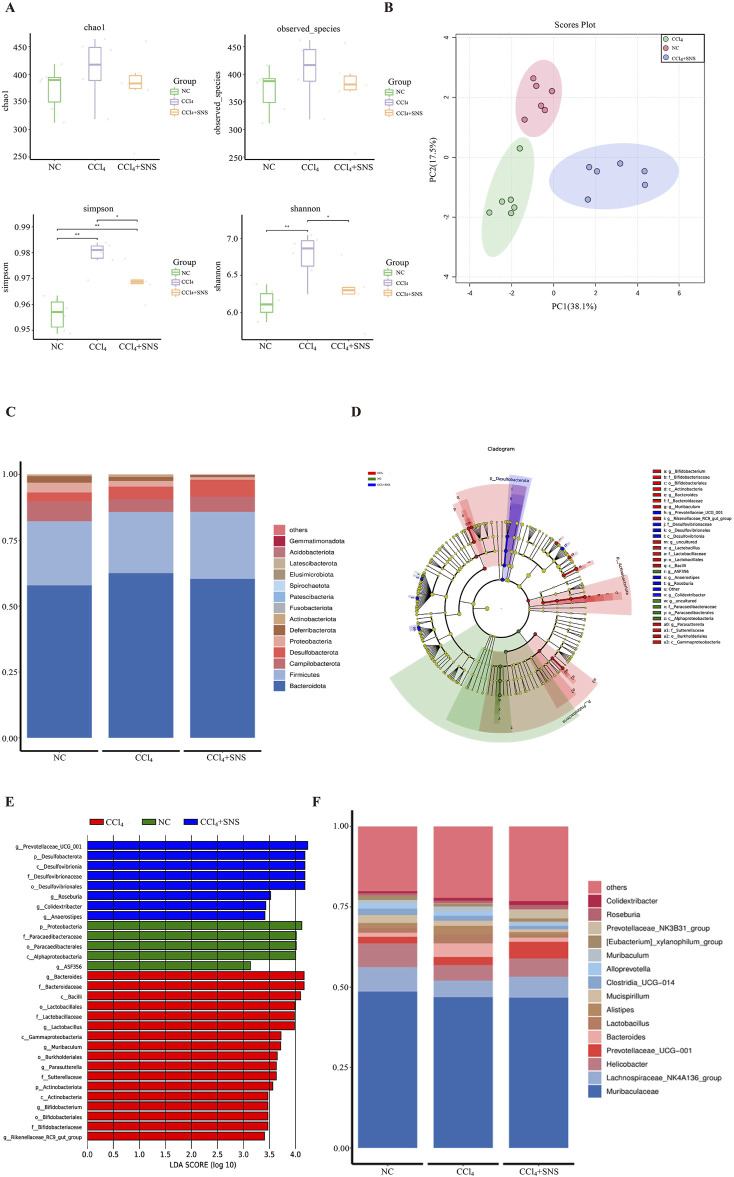
Effects of SNS on intestinal flora in CCl_4_-induced liver fibrosis mice. **(A)** Alpha diversity boxplot showing the richness and diversity of intestinal flora (observed species, Chao, Shannon, and Simpson reciprocal). **(B)** Principal component analysis (PCA) of beta diversity. **(C)** Stacked bar chart of the top 15 dominant bacterial phyla. **(D)** Taxonomic cladogram from LEfSe depicting the taxonomic associations. Each node represents a specific classification type. Red nodes represent taxonomic types with higher abundance among the three groups, and yellow nodes represent taxonomic features with insignificant differences between the groups. The diameter of the node is proportional to the relative abundance. The nodes in each layer represent phylum/class/order/family/genus from the inside to the outside. The annotations of each layer’s species labels represent phylum, class, order, family, and genus from the inside to the outside. **(E)** LDA scores calculated based on differentially enriched features among the three groups. Different colors represent different groups, and the criterion for feature selection is LDA score >3.0. **(F)** Species map of dominant bacterial species in top 15 genera. Each symbol represents an individual mouse. Data are pooled in one independent experiment with n = 6 mice per group. *P < 0.05.

To determine whether SNS treatment altered the microbiome, we performed a 16S rRNA high-throughput gene sequencing analysis of fecal bacterial DNA isolated from the mice in the NC, CCl_4_, and CCl_4_ + SNS groups. The alpha diversity of intestinal microbiota was first analyzed using a generalized linear model (Figure 1A), and SNS was found to improve the abnormal increase in alpha diversity in CCl_4_-treated mice. We then performed beta diversity analysis to generate a principal component analysis plot (PCA plot) (Figure 1B). The PCA plot showed clear cluster separation among the operational taxonomic units (OTUs), revealing different community structures among the three groups. These data suggest that the community structure of the intestinal flora is similar within the same group of mice but varies considerably among different groups. To further examine the potential differences in the composition of intestinal flora among different groups, a TOP15 phylum level species stacking diagram was created (Figure 1C). Phylum-level bacterial community composition was analyzed. All samples exhibited similar taxonomic communities. CCl_4_-induced liver fibrosis mice showed an increased abundance of Bacteroidota and a decreased abundance of Firmicutes. Treatment with SNS, however, mitigated these alterations. To further determine which bacteria were affected by the SNS treatments, high-dimensional class comparisons were performed using effect size linear discriminant analysis to evaluate the differences in bacterial community dominance among the three groups (Figures 1D, E). The results indicated an imbalance in major bacterial types in the CCl_4_ group, including *Bacteroides*, *Parasutterella*, *Muribaculum*, and *Bifidobacterium*. In contrast, these bacteria were not similarly enriched in the NC and SNS groups. Thus, SNS attenuated the abnormal enrichment of these bacterial types observed in the CCl_4_-induced liver fibrosis mice. In addition, we organized the top 15 abundances at the genus level in the intestinal microbiota of the three groups (Figure 1F). Similarly, SNS treatment decreased the abundance of genera *Bacteroides* and *Muribaculum* in CCl_4_-treated mice, which was consistent with the linear discriminant analysis (LDA) effect size (LEfSe) (Figures 1D, E). Overall, SNS treatment significantly changed the diversity and composition of the intestinal microbiota and restored CCl_4_-induced intestinal microbiota disturbances.

### 3.3 SNS decreased the serum level of propionic acid, butyric acid, and isovaleric acid

**FIGURE 2:**
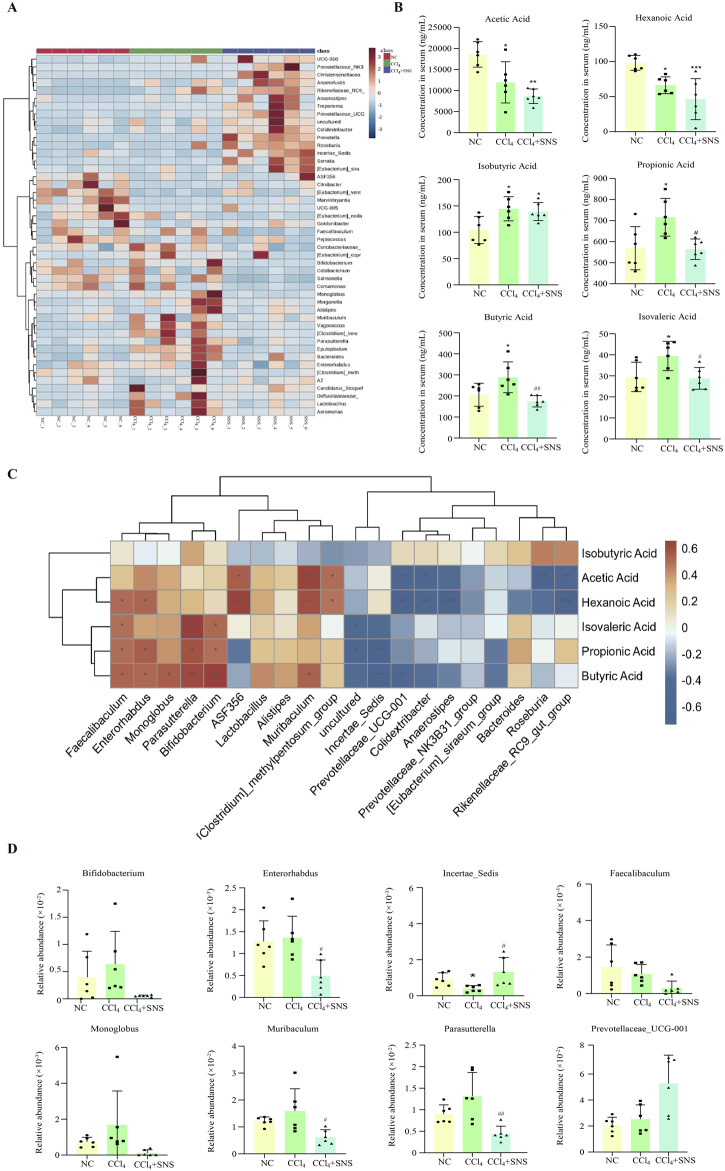
Effects of SNS on serum SCFA levels and SCFA-associated intestinal flora in CCl_4_-induced liver fibrosis mice. **(A)** Heatmap of differentially abundant features at the genus level, with blue representing less abundance, white representing intermediate abundance, and red representing the most abundance. **(B)** SCFA levels in the serum were determined by LC–MS/MS in mice. **(C)** Correlation analysis of genus-level intestinal flora and SCFAs among the three groups was performed using Spearman’s test. Red indicates a positive correlation, and blue indicates a negative correlation; *P < 0.05. **(D)** Effect of SNS on the content of SCFA-associated flora. Data are pooled in one independent experiment with n = 6 mice per group. Data are expressed as the mean ± SEM. *P < 0.05 vs. NC group; ^#^ *P* < 0.05 vs. CCl_4_ group.

Based on the linear discriminant analysis (LDA) effect size (LEfSe), notable differences were observed in the genus-level bacterial flora among the three groups. Therefore, a heatmap of the genus-level composition of the intestinal flora was generated for the three groups, while the results consistently demonstrated that SNS treatment significantly decreased the levels of *Bacteroides*, *Parasutterella*, *Muribaculum*, and *Bifidobacterium* in CCl_4_-treated mice (Figure 2A). The SCFA content was next quantitated using LC–MS/MS, and decreased levels of acetic and hexanoic acids were found in the CCl_4_ group, while this decrease in acetic and hexanoic acids was more pronounced after SNS administration (Figure 2B). Isobutyric acid levels were increased in the CCl_4_ group, while SNS treatment tends to rescue the change in isobutyric acid with no statistical significance (Figure 2B). Propionic, butyric, and isovaleric acids were increased in the CCl_4_ group, which was significantly alleviated by SNS administration (Figure 2B). To study whether changes in bacterial communities have an impact on microbial metabolic output, a correlation analysis was conducted on three groups of bacterial communities with differences in the genus level and SCFAs (Figure 2C). We observed that at the genus level, bacterial groups positively correlated with propionic acid include *Faecalibaculum*, *Enterorhabdus*, *Bifidobacterium*, and *Parasutterella*, whereas those negatively correlated include Incertae sedis. Bacterial groups positively correlated with butyric acid comprise *Faecalibaculum*, *Enterorhabdus*, *Monoglobus*, *Bifidobacterium*, *Parasutterella*, and *Muribaculum*, with negatively correlated groups being Incertae sedis and Prevotellaceae-UCG-001. Finally, bacterial groups positively correlated with isovaleric acid are *Faecalibaculum*, *Bifidobacterium*, and *Parasutterella*. Next, we constructed a histogram of the intestinal flora related to propionic, butyric, and isovaleric acids. The results showed that SNS reduced the abnormal increase in *Bifidobacterium*, *Enterorhabdus*, *Parasutterella*, *Monoglobus*, and *Muribaculum* bacteria and alleviated the decrease in Incertae sedis (Figure 2D) Typically, SNS treatment regulated the structure and composition of the intestinal bacterial community related to the contents of propionic, butyric, and isovaleric acids, which suggests that SNS improves liver fibrosis possibly by regulating the levels of propionic, butyric, and isovaleric acids metabolized by intestinal flora.

### 3.4 SNS reduced the expression of FFAR2 and FFAR3

**FIGURE 3:**
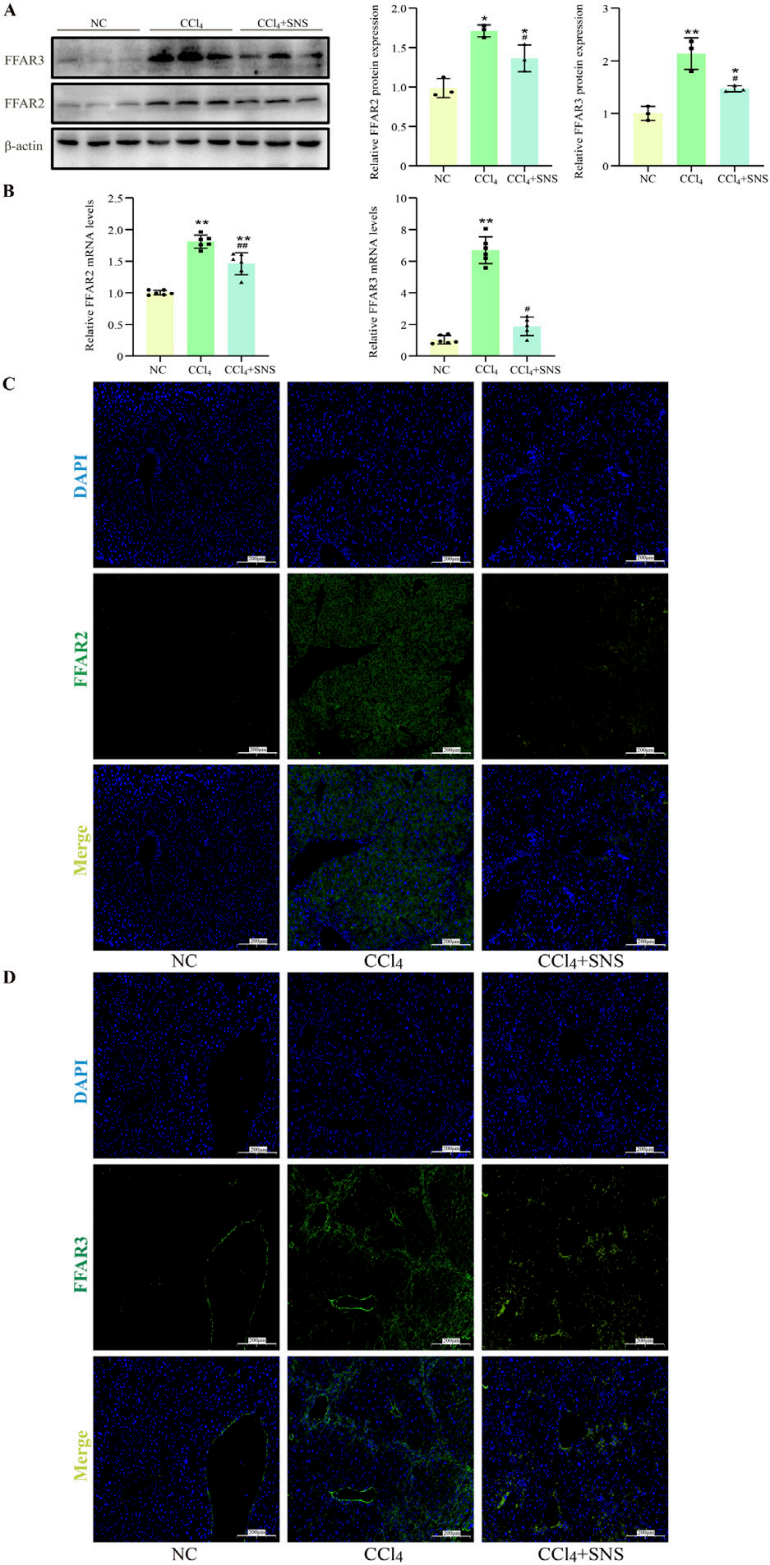
Effects of SNS on SCFA receptor expression (n = 3). **(A)** The protein expression of FFAR2 and FFAR3 in liver tissue was detected using Western blotting. **(B)** The mRNA expression of FFAR2 and FFAR3 in liver tissue was analyzed by RT-qPCR. **(C, D)** Immunofluorescence detection of the expression levels of FFAR2 and FFAR3 in liver tissue. Data were expressed as the mean ± SEM. *P < 0.05 vs. NC group; ^#^ *P* < 0.05 vs. CCl_4_ group.

Furthermore, we examined the expression of SCFA receptors FFAR2 and FFAR3 in liver tissues. Western blot results showed that SNS administration significantly reduced CCl_4_-induced hepatic expression of both FFAR2 and FFAR3 (Figure 3A), which was further consistently supported by the analyses of RT-qPCR and liver immunofluorescence (Figures 3B–D). These data suggest that SCFAs may play an important role in the progression of liver fibrosis.

### 3.5 SCFAs promoted TNF-α-induced apoptosis

**FIGURE 4:**
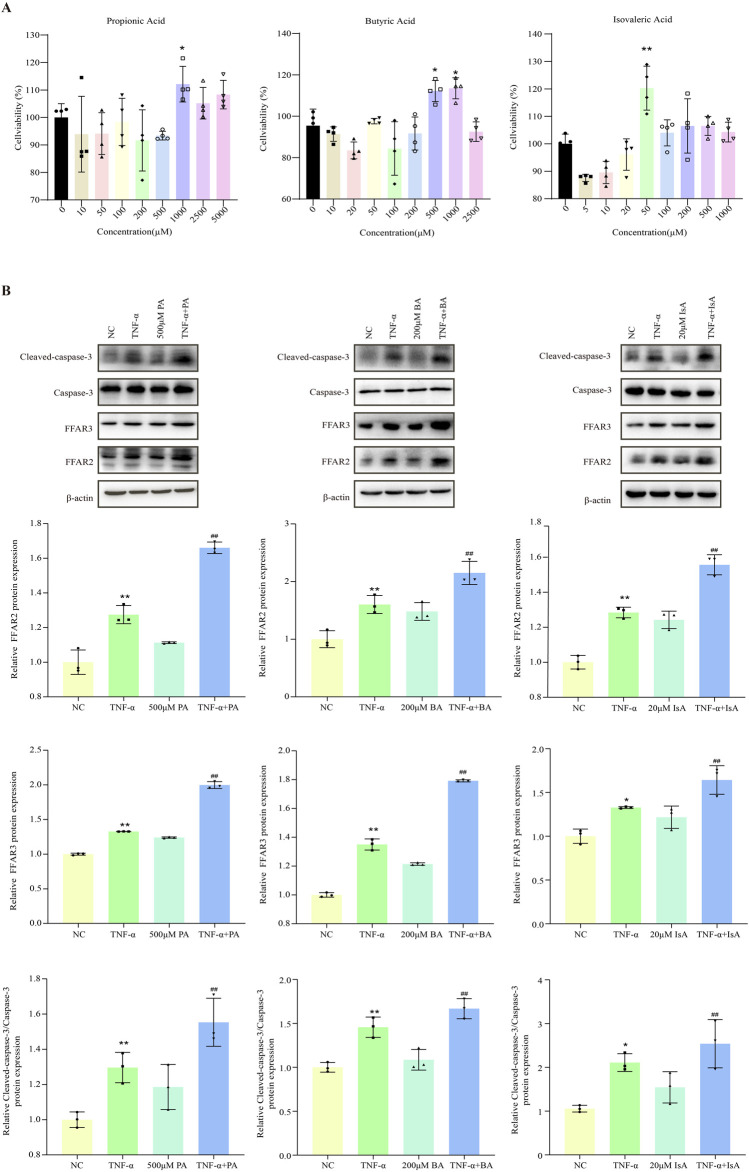
Effect of SCFAs on TNF-a-induced apoptosis in hepatocytes. **(A)** CCK8 assay of the effects of different concentrations of propionic, butyric, and isovaleric acids on HepG2 viability. **(B)** The effects of propionic, butyric, and isovaleric acids on FFAR, FFAR3, and caspase-3 protein expression in TNF-a-stimulated HepG2 cells were detected by Western blot. PA: propionic acid; BA: butyric acid; IsA: isovaleric acid. Data are expressed as the mean ± SEM. *P < 0.05 vs. NC group; ^#^ *P* < 0.05 vs. TNF-α group.

**FIGURE 5:**
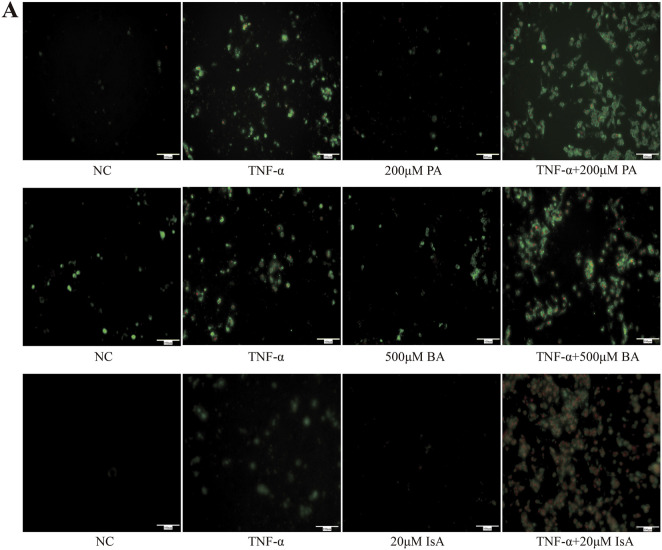
Effects of SCFA TNF-a-induced apoptosis in hepatocytes (n = 3). **(A)** Detection of apoptosis by annexin V-FITC/PI double staining. Green light represents early apoptosis, and red light represents late apoptosis.

Given the close association between SCFAs and hepatocyte injury, we further investigated the effects of various SCFAs on hepatocyte apoptosis (Singh et al., 2015; Kobayashi et al., 2018; Shao et al., 2024). HepG2 cells were first treated with increasing doses of propionic, butyric or isovaleric acids alone for 24 h, and cell viability was assessed. Cell viability was not found to be significantly decreased after treatment with 500 μM propionic acid, 200 μM butyric acid, and 20 μM isovaleric acid, suggesting that the tested SCFAs alone did not cause hepatocyte cell death (Figure 4A). Next, the effect of SCFAs on potentiating the TNF-α-induced apoptosis was further explored. FFAR2/3 expression was increased, and apoptosis was aggravated in the TNF-α-treated group compared with the control group. After the administration of propionic, butyric, and isovaleric acids, the expression of FFAR2/3 was further increased compared to that in the TNF-α group, while apoptosis was found to be significantly aggravated by CCK-8 assay (Figure 4B), which was further confirmed by the fluorescence results of apoptosis (Figure 5A).

### 3.6 Effect of SNS on pseudo-sterile mice with CCl_4_-induced liver fibrosis

**FIGURE 6:**
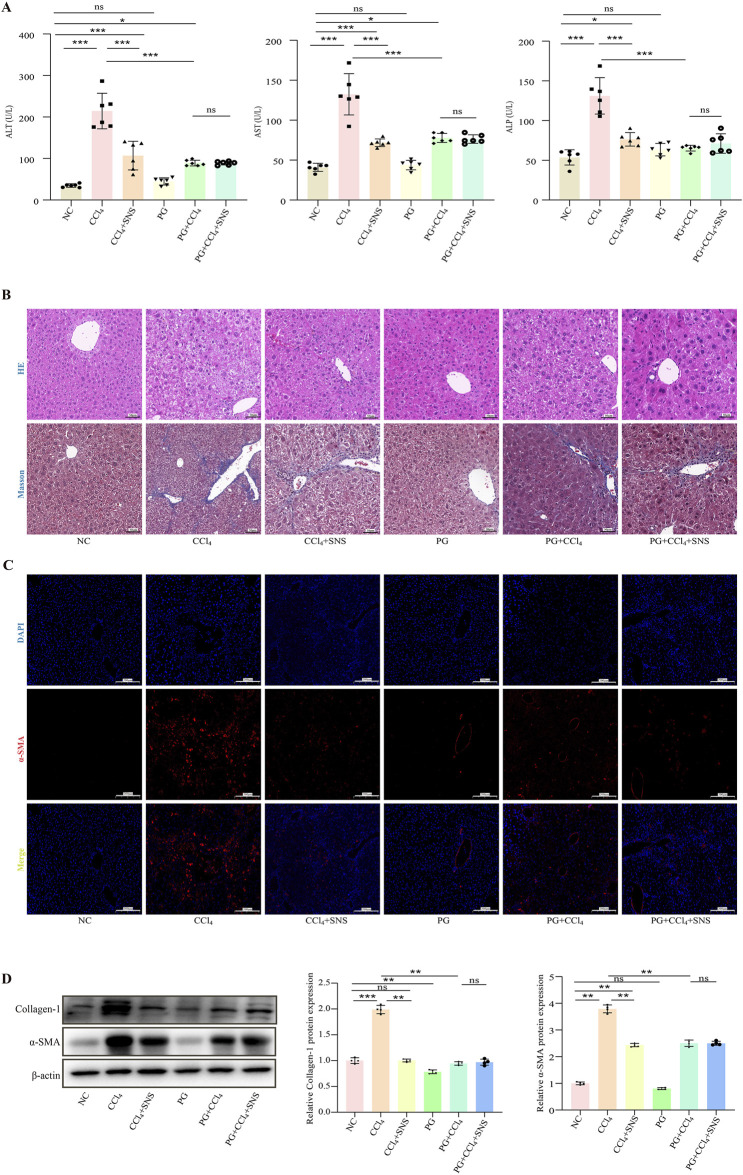
Effects of SNS on liver function and pathological changes in CCl_4_-induced liver fibrosis mice treated with an antibiotic cocktail. **(A)** Serum levels of ALT, AST, and ALP were measured using a detection kit (n = 6). **(B)** H&E and Masson staining were used to observe the pathological changes in the liver (n = 3). **(C)** α-SMA content in liver tissue samples was detected by immunofluorescence. **(D)** Collagen-1 and α-SMA protein expression in liver tissue was analyzed by Western blotting. Data are expressed as the mean ± SEM. *P < 0.05.

To investigate the potential influence of intestinal microbes on the effectiveness of SNS against liver fibrosis, intestinal microbiota were eradicated by administering a cocktail of antibiotics in drinking water. The analyses of biochemical parameters showed that the SNS treatment failed to improve CCl_4_-induced liver injury and fibrosis in the presence of antibiotics in drinking water (Figure 6A). Consistently, H&E staining showed that in the absence of intestinal flora, SNS failed to improve hepatocyte cell death and inflammatory cell infiltration in CCl_4_-treated mice. Additionally, in terms of fibrosis, Masson staining confirmed that the ameliorative effect of SNS on the formation of pseudo-leaflet and fibrotic septa was also dependent on the presence of intestinal flora (Figure 6B), which was further confirmed by the fluorescence results of α-SMA (Figure 6C) and the Western blot results (Figure 6D).

**FIGURE 7:**
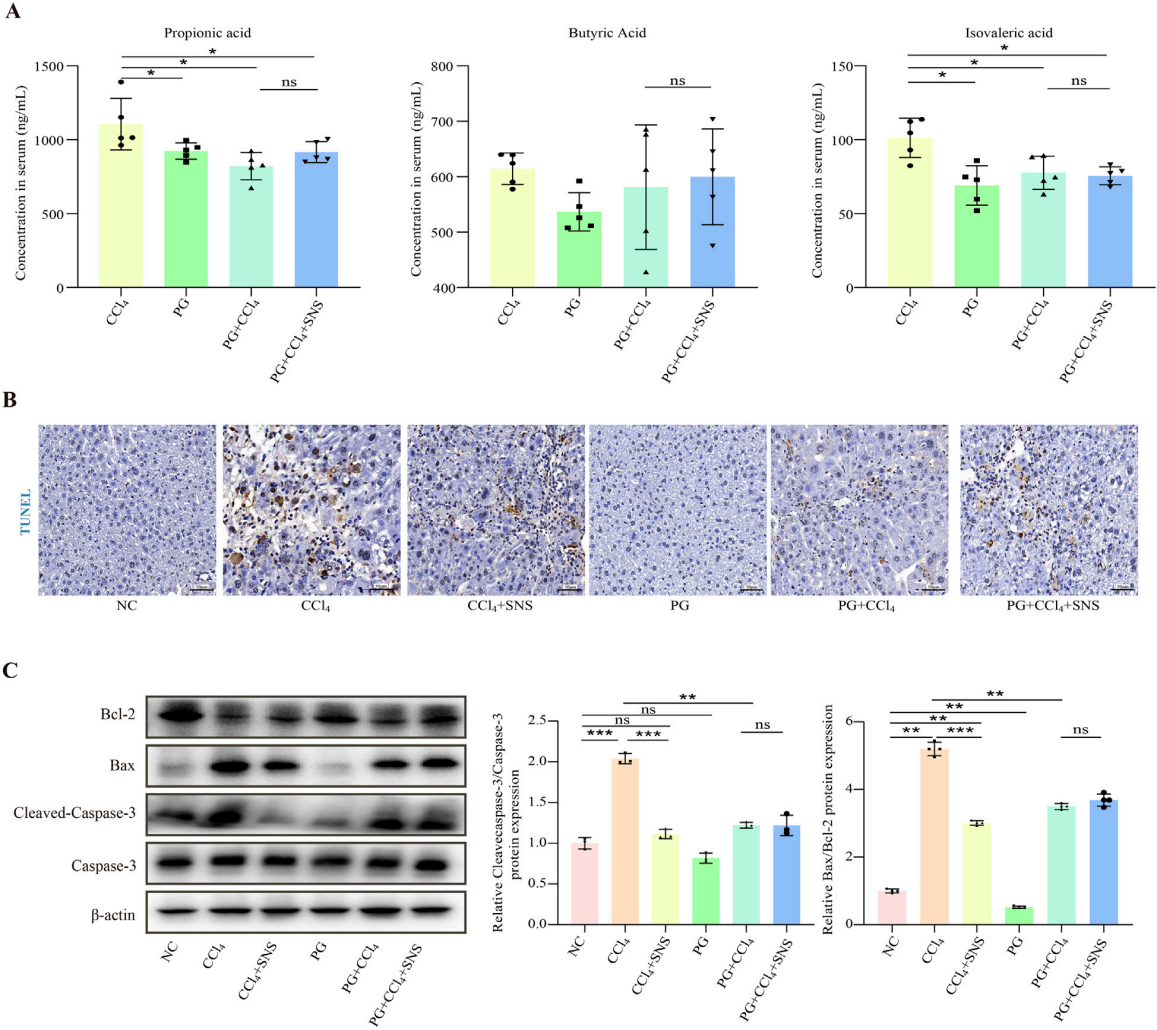
Effects of SNS on fibrosis indicators in CCl_4_-induced liver fibrosis mice treated with an antibiotic cocktail. (n = 3 or 6). **(A)** Propionic acid, butyric acid, and isovaleric acid levels in the serum were determined by LC–MS/MS in pseudo-sterile mice. **(B)** Hepatocyte apoptosis in the liver tissue of pseudo-sterile mice was assessed using TUNEL staining. **(C)** Protein expression levels of Bax, Bcl-2, and caspase-3 in the liver tissue of pseudo-sterile mice were analyzed by Western blotting. Data were expressed as the mean ± SEM. *P < 0.05.

We then asked whether SNS treatment reduces hepatocyte apoptosis by regulating the intestinal microbiota-derived SCFA metabolites and propionic, butyric, and isovaleric acid content. The results showed that mice fed antibiotics had lower levels of propionic acid, butyric acid, and isovaleric acid compared to CCl_4_-treated wild-type mice, though the difference in butyric acid was not statistically. Additionally, antibiotics mitigated the effects of SNS on propionic acid, butyric acid, and isovaleric acid (Figure 7A). Next, we detected the expression of FFAR2/3 using immunofluorescence, and the results showed that the expression of FFAR2/3 was significantly reduced in antibiotic-treated mice (Supplementary Figure S4). To further investigate the impact of antibiotic cocktail on liver cell apoptosis, we performed TUNEL staining and Western blot analyses. The results showed that apoptosis of liver cells in the PG + CCl_4_ group was significantly lower than that in the wild-type CCl_4_-induced mice (Figures 7B, C). All these data demonstrate that in the presence of antibiotics, the anti-apoptotic effect of SNS on hepatocytes may be related to the modulation of intestinal flora, particularly SCFA-associated species.

## 4 Discussion

The long-term progression of liver fibrosis leads to an exponential increase in liver-related mortality (Berumen et al., 2021). The development of effective antifibrotic drugs remains a significant challenge. The FXR agonist obeticholic acid was once considered a promising anti-liver fibrosis drug and advanced to phase III clinical trials. However, the FDA ultimately rejected it due to adverse effects, notably itching. In contrast, botanical drugs, with their multiple targets and fewer side effects, offer unique advantages in combating liver fibrosis. SNS is a traditional Chinese medicine prescription primarily used for treating chronic hepatitis and liver–stomach disharmony (Zhu et al., 2019). Recently, SNS has been employed in treating liver fibrosis (Wang et al., 2021), but its pharmacological mechanism remains unclear.

Hepatocytes, the primary liver cells, are pivotal in the onset of liver diseases. Hepatocyte apoptosis is a key pathological feature in liver fibrosis, driving the fibrotic process through the activation of Fas death receptors and the release of apoptotic bodies. Thus, targeting hepatocyte apoptosis represents a promising strategy for treating liver fibrosis (Calès, 1998; Roehlen et al., 2020; Khanam et al., 2021; Guo et al., 2022). Recent studies suggest that this approach is a common pharmacological mechanism for several botanical drugs, such as Yinchenhao decoction, and extracts from *Zygophyllum album* leaves and *Kadsura heteroclita* stem (Cai et al., 2019; Feriani et al., 2020; Yu et al., 2021). Both our previous study and the current manuscript confirm that SNS effectively ameliorates hepatocyte apoptosis in mice with CCl_4_-induced liver fibrosis. This may be an important pharmacological mechanism for the anti-hepatic fibrosis effect of SNS (Jiang et al., 2023).

The liver–gut axis is essential for maintaining normal liver function, with intestinal flora homeostasis playing a crucial role (Albillos et al., 2020; Tilg et al., 2022). Imbalances in the intestinal flora are characteristic in patients with liver fibrosis and cirrhosis (Nishimura et al., 2021; Li S. et al., 2022; Huang et al., 2023; Zhang et al., 2023). These imbalances accelerate liver fibrosis due to immune regulation disturbances and toxic metabolites (Roehlen et al., 2020; Nie et al., 2021; Nishimura et al., 2021). Increasing evidence suggests a close association between intestinal flora disorders and hepatocyte apoptosis (Wan et al., 2019; Nishimura et al., 2021; Wang et al., 2022). The four dominant intestinal phyla are Bacteroidota, Firmicutes, Proteobacteria, and Actinobacteria. Studies have shown that Bacteroidota are significantly higher in patients with liver damage compared to healthy individuals (de Faria Ghetti et al., 2018; Vallianou et al., 2021). Similar findings have been reported in conditions such as non-alcoholic fatty liver disease, liver fibrosis, and cirrhosis. In contrast, the abundance of Firmicutes was reduced in patients with cirrhosis (Acharya and Bajaj, 2017). Consistent with these findings, we observed increased Bacteroidota and decreased Firmicutes abundance in CCl_4_-induced hepatic fibrosis mice.

Recent studies have demonstrated the modulation of intestinal flora by SNS, which may be an important mechanism by which SNS ameliorates colonic injury and non-alcoholic fatty liver disease (Zhu et al., 2019; Xu et al., 2023). Using pseudo-sterile mice induced by antibiotic cocktail therapy, we observed reduced hepatic fibrosis and hepatocyte apoptosis. Notably, antibiotics negated the anti-apoptotic effect of SNS, suggesting that intestinal flora disturbances contribute to hepatocyte apoptosis in CCl_4_-induced hepatic fibrosis mice. This highlights flora balance restoration as a potential target for SNS’s anti-apoptotic activity. We found that SNS can restore the levels of Bacteroidota and Firmicutes at the phylum level, which were altered by CCl_4_ induction. The dysregulation characterized by increased Bacteroidota and decreased Firmicutes levels is a hallmark of gut microbiota imbalance in cirrhosis, correlating with elevated endotoxin levels observed in patients with hepatic encephalopathy and spontaneous bacterial peritonitis in cirrhosis. Furthermore, insulin therapy in diabetic cirrhotic patients has been noted to influence Bacteroidota and Firmicutes abundance. Importantly, supplementation with Firmicutes has shown beneficial effects in cirrhosis (Acharya and Bajaj, 2017). Consistent with our findings, animal studies have demonstrated that curcumin protects against CCl_4_-induced liver fibrosis by restoring Bacteroidota and Firmicutes homeostasis (Zheng et al., 2022). At the genus level, SNS mitigated the CCl_4_-induced elevations of *Bacteroides*, *Parasutterella*, *Muribaculum*, *Enterorhabdus*, *Monoglobus*, and *Bifidobacterium*. Elevated levels of *Bacteroides*, *Parasutterella*, and *Muribaculum* have been observed in both alcoholic and non-alcoholic liver disease models, with pharmacological interventions restoring the homeostasis of these bacterial colonies (Yang et al., 2019; Lin et al., 2023a; Li et al., 2024). In CCl_4_-induced liver fibrosis mice, metformin normalized *Bacteroides* and *Parasutterella* levels (Kong et al., 2024), while the Yi–Qi–Jian–Pi formula restored *Muribaculum* homeostasis (Yang et al., 2024), which are consistent with our findings. Moreover, the CCl_4_-induced increase in *Enterorhabdus* abundance positively correlates with the upregulation of liver lipid metabolism genes (Zhao et al., 2024). Additionally, Yusufu et al. suggest that *Enterorhabdus* may be involved in systemic inflammation due to a low tryptophan diet (Yusufu et al., 2021). In children with biliary atresia, increased abundance of *Monoglobus* was observed (Yang et al., 2022).

The role of *Bifidobacterium* in liver fibrosis is controversial (Hizo and Rampelotto, 2023). It is generally considered a beneficial bacterium, typically reduced in cirrhotic patients, and exogenous supplementation of *Bifidobacterium* is considered beneficial for hepatic fibrosis (Hizo and Rampelotto, 2023). However, other clinical studies have found elevated levels of *Bifidobacterium* in patients with cirrhosis (Dubinkina et al., 2017; Kajihara et al., 2019). Numerous clinical studies have shown that supplementing with probiotics, including *Bifidobacterium*, *Lactobacillus acidophilus,* and *Enterococcus faecalis*, does not significantly improve hepatic fibrosis (Zhou et al., 2021; Escouto et al., 2023). This controversy may be attributed to the inclusion of patients with varying stages of liver fibrosis in different studies and the administration of antibiotics, which disrupt intestinal microbiota homeostasis. Additionally, the complex interactions within the intestinal microbiota make it challenging to achieve consistent experimental results (Escouto et al., 2023). These findings underscore the need for in-depth mechanistic studies, such as fecal microbiota transplantation (FMT), to further clarify the roles of different microbial communities.

SCFAs are derived from bacterial fermentation and breakdown of dietary fibers as well as peptides and proteins in the gut and are important substrates and signaling molecules for maintaining body functions. For example, butyrate is a substrate for energy metabolism in colonic cells, while propionate promotes gluconeogenesis in the liver (Wang et al., 2023). Most studies have concluded that supplementation with SCFAs is beneficial, but this is not absolute and is dependent on the concentration of SCFAs and the microenvironment of the cells (Shao et al., 2024). However, the latest clinical study suggests an abnormality in the concentration of SCFAs in patients with cirrhosis: serum propionic acid and butyric acid levels in patients with cirrhosis were significantly elevated; isovaleric acid was mildly elevated in the serum, but there was no statistically significant difference (Wang et al., 2023), which is similar to the results of our study. Wang et al. suggested a correlation between serum SCFAs and cirrhosis, specifically highlighting the role of isovaleric acid, despite its low concentration.

Previous studies have confirmed that SCFAs are involved in the apoptosis of various cells. Butyrate induces apoptosis in breast cancer and colorectal cancer cells by downregulating miR-17-92a levels and inhibiting histone deacetylase (HDAC) (Hersi et al., 2022; Rekha et al., 2024). Additionally, research has indicated that SCFAs are associated with hepatocyte apoptosis. In an arsenic-induced injury model, elevated levels of fecal propionate, butyrate, and isovalerate were observed, accompanied by hepatocyte apoptosis (Shao et al., 2024). Propionate, in combination with cisplatin, induces TNF-α expression and promotes apoptosis in HepG2 cells through FFAR3 activation and HDAC inhibition (Kobayashi et al., 2018). In our *in vitro* experiments, propionic acid, butyric acid, and isovaleric acid alone had no significant effect on HepG2 cells, but they could synergize with TNF-α to induce apoptosis. Additionally, we observed increased expression of FFAR2 and FFAR3 in both fibrotic mice and HepG2 models, suggesting that the apoptosis induced by these SCFAs may be related to receptor activation. Although numerous studies have demonstrated the protective effects of SCFAs against apoptosis (Pingitore et al., 2019; Guo et al., 2023; Saikachain et al., 2023), variations in the cellular microenvironment and SCFA concentrations can yield different experimental outcomes. Consequently, further research is required to confirm the effects of SCFAs on hepatocytes.

Our experiments still have some limitations. Although SNS appears to restore gut microbiota homeostasis, we did not assess microbiota changes in pseudo-sterile mice or conduct fecal microbiota transplantation (FMT) experiments. Consequently, the specific target microbiota of SNS and its mechanisms for regulating microbiota homeostasis remain unclear. Additionally, although SCFAs were found to enhance TNF-α-induced HepG2 apoptosis, the precise underlying mechanisms are not fully elucidated. Further research is needed to determine whether apoptosis is linked to FFAR2, FFAR3, or HDAC activity.

Nevertheless, our experiments confirm that SNS’s anti-hepatic fibrosis effect is associated with restoring intestinal flora homeostasis and regulating SCFA metabolism. In CCl_4_-induced hepatic fibrosis mice, SNS effectively reduced elevated serum levels of propionic acid, butyric acid, and isovaleric acid. These SCFAs, which can synergize with TNF-α to induce HepG2 apoptosis, may act through FFAR2 and FFAR3 activation. Further studies are needed to validate these mechanisms. Overall, restoring intestinal flora homeostasis remains a key target of SNS’s anti-hepatic fibrosis effect.

## Data Availability

The datasets presented in this study can be found in online repositories. The names of the repository/repositories and accession number(s) can be found below: https://www.ncbi.nlm.nih.gov/, PRJNA1088426.

## Abbreviations

SNS, Si-Ni-san; SCFAs, short-chain fatty acids; CCl_4_, carbon tetrachloride; AST, aspartate transferase; ALT, alanine transaminase; ALP, alkaline phosphatase; H&E, hematoxylin–eosin; α-SMA, α-smooth muscle actin; ActD, actinomycin D; TNF-α, tumor necrosis factor α; CCK-8, Cell Counting Kit-8; TUNEL, terminal deoxynucleotidyl transferase dUTP nick end labeling; OTUs, operational taxonomic units; PCA, principal component analysis; LEFSe, linear discriminant analysis effect size.
