# Corrigendum: Sini san regulates intestinal flora and short-chain fatty acids to ameliorate hepatocyte apoptosis and relieve CCl_4_-induced liver fibrosis in mice

**DOI:** 10.3389/fphar.2024.1516840

**Published:** 2024-11-18

**Authors:** Qiong Wu, Fangsi Zhu, Yu Yao, Luyun Chen, Yijie Ding, Yong Su, Chaoliang Ge

**Affiliations:** ^1^ School of Pharmacy, The First Affiliated Hospital of Anhui Medical University, Anhui Medical University, Hefei, China; ^2^ Department of Pharmacy, Anhui No. 2 Provincial People’s Hospital, Hefei, Anhui, China; ^3^ Department of Pharmacy, The First Affiliated Hospital of Anhui Medical University, Hefei, Anhui, China

**Keywords:** Si-Ni-San, intestinal flora, short-chain fatty acids, hepatocyte apoptosis, liver fibrosis, pseudo germ-free mice

In the published article, there was an error regarding the **Affiliation** for **Chaoliang Ge**. As well as having affiliation **3**, they should also have ‘School of Pharmacy, The First Affiliated Hospital of Anhui Medical University, Anhui Medical University, Hefei, China.’

The authors apologize for this error and state that this does not change the scientific conclusions of the article in any way. The original article has been updated.

