# Supplementary material for: Sini san regulates intestinal flora and short-chain fatty acids to ameliorate hepatocyte apoptosis and relieve CCl4-induced liver fibrosis in mice
Source: Front Pharmacol. 2024 Aug 30;15:1408459. doi: 10.3389/fphar.2024.1408459 (PMC11392872; doi:10.3389/fphar.2024.1408459)
Supplement: Supplementary file 1 [file DataSheet1.docx]

Supplemental Table S1. Primer sequences

| Gene (mouse) | Primer sequence (5' to 3') |
| --- | --- |
| *α-SMA* | Forward：GTCCCAGACATCAGGGAGTAA |
|  | Reverse： TCGGATACTTCAGCGTCAGGA |
| *Collagen-1* | Forward：TAGGCCATTGTGTATGCAGC |
|  | Reverse：ACATGTTCAGCTTTGTGGACC |
| *FFAR2* | Forward：GGTGGAGGCTGTGGTGTT |
|  | Reverse：GCATAGAGGAGGCAGGATT |
| *FFAR3* | Forward：CTCATCACCAGCTACTGCCG |
|  | Reverse：AATTCAGGGTGCTGAGGAGC |
| *β-actin* | Forward：ACCTCTATGCCAACACAGTG |
|  | Reverse：GGACTCATCGTACTCCTGCT |

**Figure S1**

**
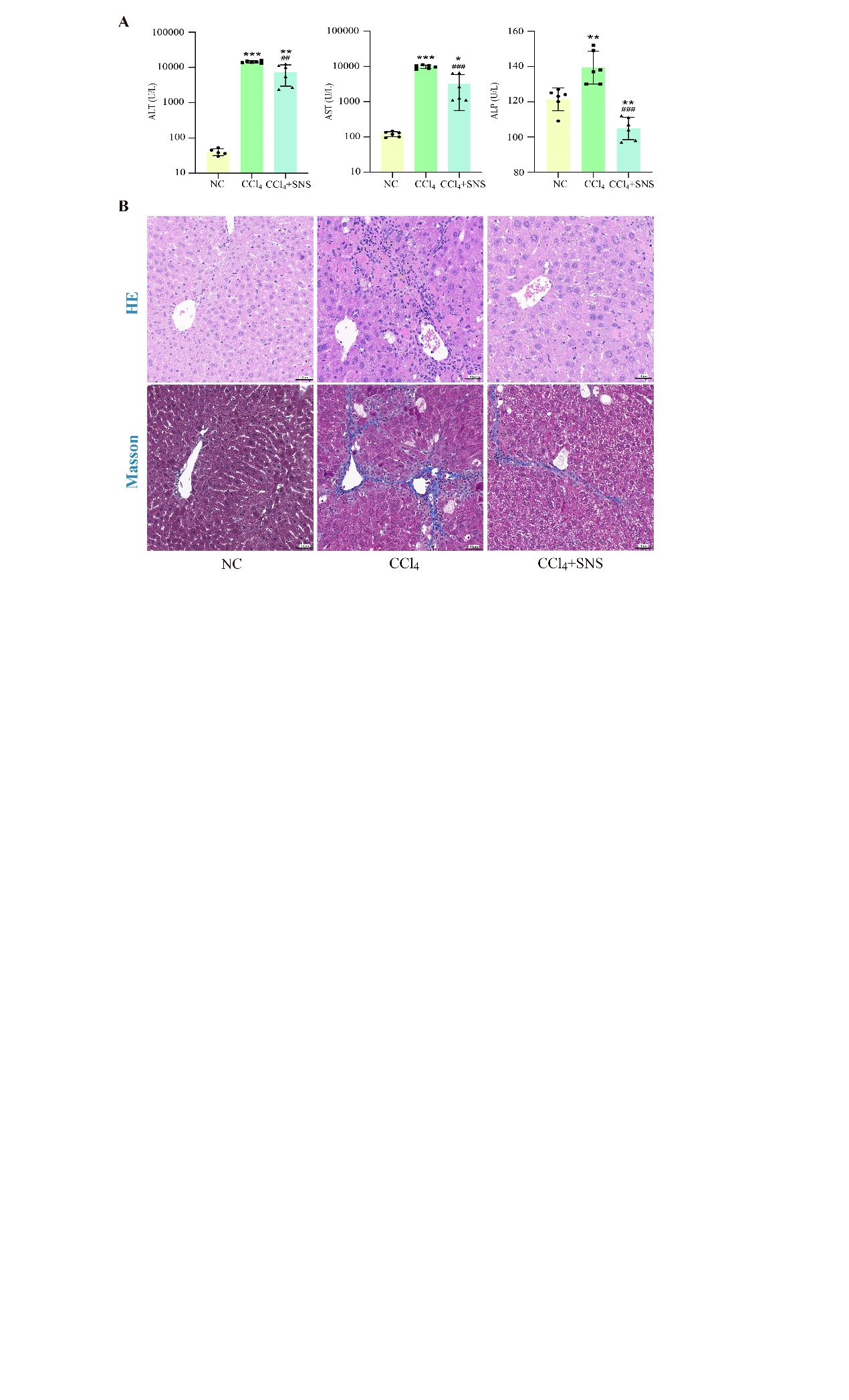
**

**Supplemental Figure S1** Effect of SNS on liver function and pathological changes in CCl4-induced liver fibrosis mice. (A) Serum levels of ALT, AST, and ALP were measured using a detection kit (n=6). (B) H&E and Masson staining were used to observe the pathological changes of the liver (n=3). Data were expressed as the mean ± SEM. **P* < 0.05 vs. NC group; ^#^*P* < 0.05 vs. CCl_4_ group.

**Figure S2**


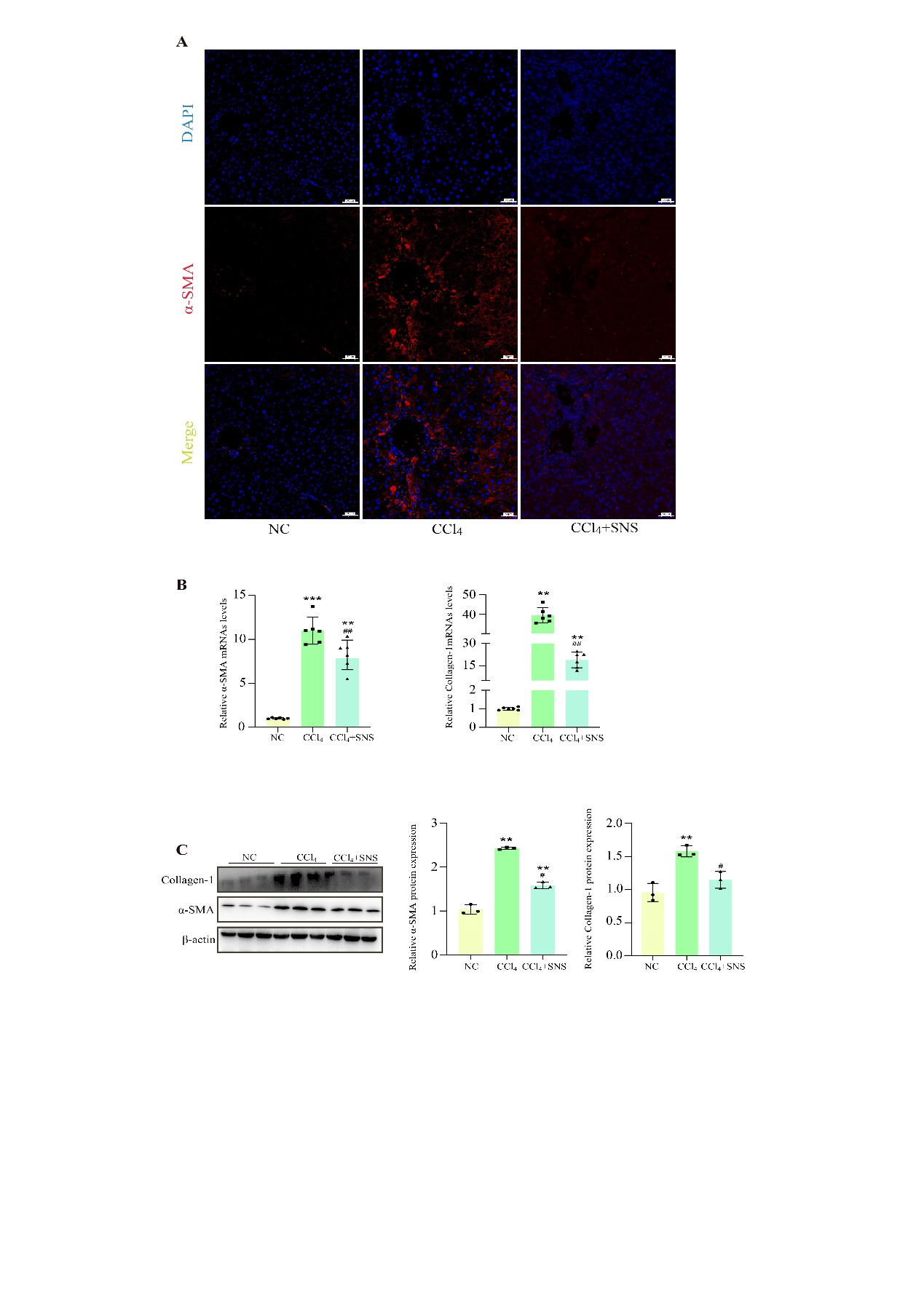


**Supplementary Figure S2** Effect of SNS on fibrosis indicators in CCl_4_-induced liver fibrosis mice. (n = 3). (A) α-SMA content in liver tissue samples was detected by immunofluorescence. (B) Collagen-1 and α-SMA mRNA expression levels in liver tissue were measured using RT-qPCR. (C) Collagen-1 and α-SMA protein expression in liver tissue was analyzed by Western blotting. Data were expressed as the mean ± SEM. **P* < 0.05 vs. NC group; ^#^*P* < 0.05 vs. CCl_4_ group.

**Figure S3**

**
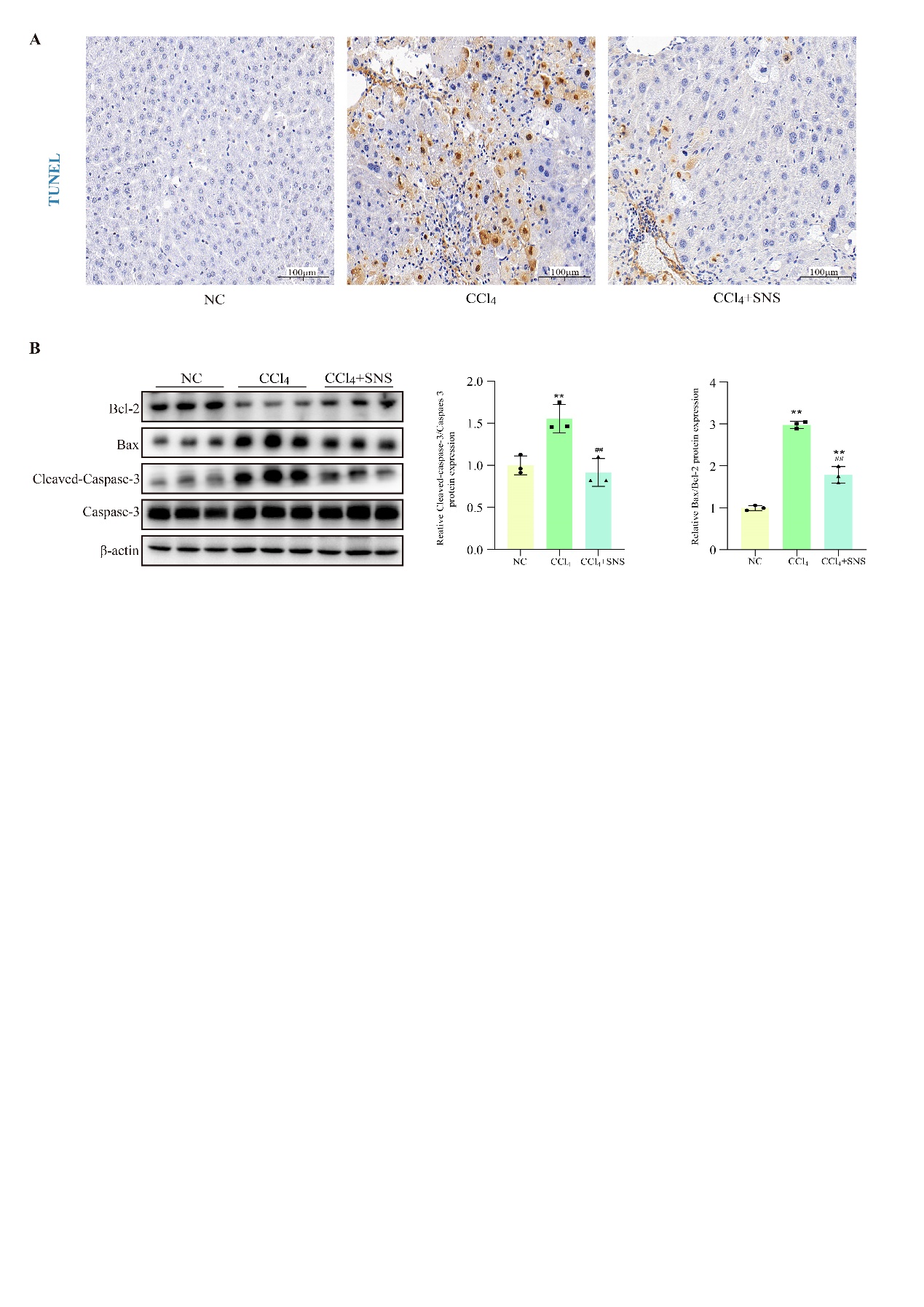
**

**Supplemental Figure S3** The effect of SNS on hepatocyte apoptosis in CCl_4_-induced liver fibrosis mice (n=3). (A) Hepatocyte apoptosis in liver tissue was assessed using TUNEL staining. (B) Protein expression levels of Bax, Bcl-2, and Caspase-3 in liver tissue were analyzed by Western blotting. Data were expressed as the mean ± SEM. **P* < 0.05 vs. NC group; ^#^*P* < 0.05 vs. CCl_4_ group.

**Figure S4**


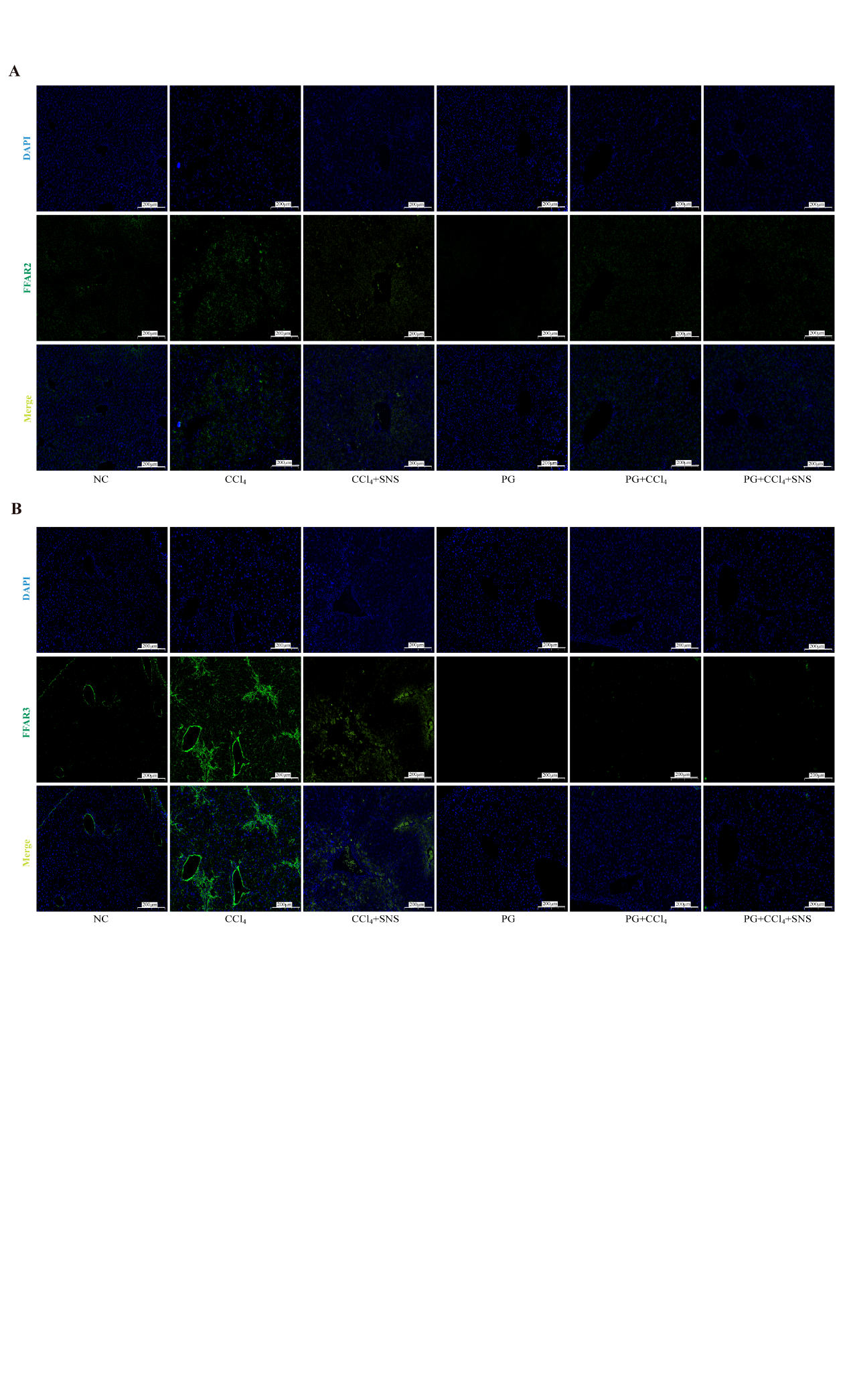


**Supplemental Figure S4.** Effect of SNS on the protein expression of FFAR2 and FFAR3 in CCl_4_-induced liver fibrosis mice treated with an antibiotic cocktail (n=3). (A) Protein expression of FFAR2 in liver tissue of pseudo-sterile mice was detected by immunofluorescence. (B) Protein expression of FFAR3 in liver tissue of pseudo-sterile mice was detected by immunofluorescence.
